# The process of developing and implementing a telephone-based peer support program for postpartum depression: evidence from two randomized controlled trials

**DOI:** 10.1186/1745-6215-15-131

**Published:** 2014-04-17

**Authors:** Cindy-Lee Dennis

**Affiliations:** 1 Lawrence S. Bloomberg Faculty of Nursing, University of Toronto, 155 College Street, Suite 130, Toronto, ON M5T 1P8, Canada; 2 Women’s College Hospital, Women’s College Research Institute, 790 Bay Street, Toronto, ON M5G 1N8, Canada

**Keywords:** Peer support, Postpartum depression, Prevention, Randomized controlled trial

## Abstract

**Background:**

A randomized controlled trial evaluated the effect of telephone-based peer support on preventing postpartum depression (PPD) among high-risk mothers. The results indicated that support provided by peer volunteers may be an effective preventative strategy. The purpose of this paper is to outline the process of developing, implementing, maintaining, and evaluating the peer support program that we used in this PPD prevention trial.

**Methods:**

The peer support program had been used successfully in a pilot trial and a previous breastfeeding peer support trial. Based on our experience and lessons learned, we developed a 4-phase, 12-step approach so that the peer support model could be copied and used by different health providers in various settings. We will use the PPD prevention trial to demonstrate the suggested steps.

**Results:**

The trial aim to prevent the onset of PPD was established. Peer volunteers who previously experienced and recovered from self-reported PPD were recruited and attended a four-hour training session. Volunteers were screened and those identified as appropriate to provide support to postpartum mothers were selected. Women who scored more than 9 on the Edinburgh Postnatal Depression Scale within the first two weeks after childbirth were recruited to participate in the trial and proactive, individualized, telephone-based peer support (mother-to-mother) was provided to those randomized to the intervention group. Peer volunteers maintained the intervention, supported other volunteers, and evaluated the telephone-based support program. Possible negative effects of the intervention were assessed. An in-depth assessment of maternal perspectives of the program at 12 weeks postpartum was performed.

**Conclusions:**

The 4-phase, 12-step approach delineated in this paper provides clear and concise guidelines for health professionals to follow in creating and implementing community-based, peer-support interventions with the potential to prevent PPD.

**Trial registration:**

Current Controlled Trials ISRCTN68337727.

## Background

Depression is among the most disabling disorders for women in their childbearing years. For women aged 15 to 44, it is the leading cause of non-obstetric hospitalization among women in the United States [1]. Postpartum depression (PPD) is defined as a major depressive disorder in the Diagnostic and Statistical Manual of Mental Disorders (DSM-IV), with a specifier of postpartum onset within four weeks after childbirth [2]. Among health care providers and researchers, PPD is often considered when a depressive episode occurs within the first year after childbirth [1]. A recent meta-analysis reported that as many as 19.2% of mothers may have a depressive episode during the first three months postpartum with 7.1% experiencing major depression [3].

Unfortunately, PPD has negative health consequences for the mother, child and family. Women who have suffered from PPD are twice as likely to experience future episodes of depression over a five-year period [4]. PPD is associated with impaired mother-infant interactions that include maternal withdrawal, disengagement, intrusion, and hostility [5,6]. Women with PPD may also be less likely to initiate or maintain breastfeeding; depressive symptoms commonly precede the early cessation of breastfeeding [7,8]. PPD can also impair child development [9,10]. Infant and child outcomes that are associated with PPD include a higher incidence of excessive infant crying or colic, sleep problems, and temperamental difficulties [11,12]. PPD is also linked to poor cognitive functioning, behavioral inhibition, and emotional maladjustment in infants and children [13-15]. Despite these negative outcomes, PPD often remains undetected due to maternal reluctance to disclose symptoms of depression and seek treatment even when they are in frequent contact with health professionals [16].

Detailed analysis of social support variables in predictive studies clearly suggest that inadequate support is a risk factor for the development of PPD [17-19]. Indeed, the risk of PPD is increased by various support deficiencies including: (a) not having someone to talk openly with who has shared a similar problem; (b) lacking an intimate confidant or friend; (c) not receiving support without having to ask for it; and (d) feeling socially isolated [17,19,20]. When depressed mothers participating in one population-based study were asked why they thought they were depressed, lack of support and feeling isolated were the most common responses [21]. When asked what advice they would give to new mothers currently suffering from PPD, the most common suggestion was to find someone to talk to. Not surprisingly, scores on self-report measures that are available to screen for PPD, such as the Edinburgh Postnatal Depression Scale (EPDS) [22], are significantly correlated to maternal perceptions of support from other women with children [23]. Thus, the provision of support from an experienced mother may be a simple intervention with the potential to prevent PPD.

In other areas of health peer support interventions have been successful in facilitating psychological adjustment, aiding recovery from traumatic experiences, and even extending life for individuals with serious chronic diseases [24]. In light of this evidence, a randomized controlled trial was conducted to evaluate the effect of telephone-based peer support in the prevention of PPD among women identified as high risk within the first two weeks postpartum [25]. In this trial, peer support consisted of individualized, mother-to-mother support initiated within 48 to 72 hours of randomization and provided by a volunteer recruited from the community. It was hypothesized that fewer women who received peer support would show evidence of PPD at 12 weeks postpartum than those who did not receive peer support. After screening more than 20,000 women who had just given birth, 701 eligible mothers were recruited and randomized to control (usual care, *n* = 352) and intervention (usual care plus telephone peer support, *n* = 349) groups. Participant follow-up rates for the 12 and 24 weeks postpartum assessments were greater than 85%. At 12 weeks postpartum, significantly fewer women randomized to the intervention group (13.5%) had an EPDS score >12 when compared with those in the control group (24.8%; odds ratio 2:1, 95% confidence interval 1.38 to 3.20; *χ*^2^ = 12.5, *P* < 0.001) [25]. These results indicate that support provided by peer volunteers may be an effective strategy for preventing PPD and its impact on mothers and their children. The purpose of this paper is to outline the process of developing, implementing, maintaining, and evaluating the peer support program that we used in this PPD prevention trial.

## Methods

The peer support program had been used successfully in a pilot trial to evaluate the effect of peer support on depressive symptomatology among mothers identified as high-risk for PPD [26] and a previous randomized controlled trial to evaluate the effect of peer support on breastfeeding duration among primiparous women [27]. Based on our experience and lessons learned, we have developed the following 4-phase, 12-step approach so that the peer support model can be copied and used by different health providers in various settings (Table 1). We will use the PPD prevention trial to demonstrate the suggested steps.

**Table 1:** Description of the four phases of a peer support program

PHASE I: DEVELOP THE INTERVENTION	
Step 1)	Establish the project aim
Step 2)	Recruit peer volunteers
Step 3)	Traini peer volunteers
Step 4)	Screen/accept peer volunteers
PHASE II: IMPLEMENT THE INTERVENTION	
Step 5)	Recruit postpartum women to participate
Step 6)	Initiate contact between peer volunteers and postpartum women
Step 7)	Provide peer support
PHASE III: MAINTAIN THE INTERVENTION	
Step 8)	Monitor the intervention initiation and implementation
Step 9)	Support the peer volunteers
Step 10)	Review participant safety and negative effects of the intervention
PHASE IV: EVALUATE THE PEER SUPPORT PROGRAM	
Step 11)	Evaluation of the telephone support program by peer volunteers
Step 12)	Evaluation of the telephone support program by participants

## Results

### Phase I: develop the intervention

#### Step 1: establish the aim of the project

Peer support interventions have been used for a variety of health-related topics including transitional stressors (such as prenatal care, loss of a spouse or breastfeeding), chronic disease (for example diabetes or renal failure), and health promotion (including STD prevention, cancer screening, and conflict resolution) [28]. An important first step in the development of a peer support program is to specify a focused target condition in a specific community. In this program, we aimed to prevent the onset of PPD in women identified in the immediate postpartum period as being at high risk. This study was approved by the University of Toronto ethics committee and ethical review boards of participating health regions. Informed consent was given by all participants, both the peer volunteers and the at-risk mothers to whom they provided support.

#### Step 2: recruit peer volunteers

A ‘peer’ is someone who possesses both similar characteristics as the target population and experiential knowledge of a specific behaviour or stressor [28]. We chose to recruit peer volunteers with a self-reported history of PPD and match them with new mothers based on geographic location and ethnicity (if desired by the mother). Due to the size of the program, a fulltime, paid volunteer coordinator was hired to: (a) organize the recruitment of peer volunteers; (b) obtain informed consent; (c) conduct peer volunteer training sessions; (d) match postpartum women with an appropriate peer volunteer; (e) assist in the organization of peer volunteer meetings and discussions on an electronic bulletin board; (f) monitor the intervention implementation; and (g) provide support to peer volunteers as required. The peer volunteer coordinator had previous experience working with mothers. No formal qualifications were required other than having communication and organization skills and being reliable.

The project setting included seven health regions across Ontario, Canada. Between September 2004 and June 2006, flyers were distributed and newspaper ads were placed in the participating regions to enlist peer volunteers. Peer volunteers had to have had PPD in the past ten years. They were not assessed for current mental health status using a questionnaire but were asked if they were currently experiencing depression. They were also monitored for depressive symptoms and their ability to talk about their PPD experience during the training. To protect confidentiality, volunteers were assigned with a code number for all of their questionnaires and participant matching. Due to diverse recruitment strategies, we had no difficulty recruiting peer volunteers, however, recruitment was continuous due to volunteer turnover.

Two hundred and four women from the community volunteered, were able to speak and understand English, had a self-reported history of and recovery from PPD. The majority of the peer volunteers who participated in the trial were married (*n* = 168, 82.4%) and had some postsecondary education (*n* = 187, 91.7%); 61% (*n* = 125), were multiparous and 41.7% (*n* = 85) were employed outside of the home. Over half (*n* = 93, 54%) of the peer volunteers self-reported their nationality as non-Canadian.

#### Step 3: train peer volunteers

Helpful mechanisms of peer interventions include the provision of emotional support (expression of caring, encouragement and attentive listening), informational support (such as advice, suggestions, and factual input) and appraisal support (for example the communication of optimism, assistance to endure frustration, and encouragement to persist in problem resolution) [28]. All peer volunteers participated in a four-hour training session. The training sessions were comprised of small groups of up to eight volunteers at a time. The focus of the training was to develop the skills required to provide effective telephone-based peer support and to make referrals to health professionals as necessary; role-playing and strategizing were important components of the training sessions. Peer volunteers were also able to practice new skills in the training in a role modelling session. A ‘Mothers Helping Mothers with Postpartum Depression’ manual was developed, pilot-tested and distributed to all new peer volunteers [26]. This 121-page training manual outlined the professional services available for referral and covered relevant topics including: (a) introduction to peer support; (b) potential benefits of peer support; (c) relationship development; (d) techniques for effective telephone support; (e) general PPD information, (f) the helping process; and (g) referral to professional services (Table 2). The manual provided the basis for the training and was to be referred to by peer volunteers on their own at home when matched with a new mother. During the training sessions all peer volunteers were given several activity logs, which included postage-paid, addressed envelopes to document all peer volunteer activities to 12 weeks postpartum. In the training session, all peer volunteers were provided with clear guidelines regarding maternal self-harm thoughts and when to refer mothers to professional health services. Peer volunteers were requested to maintain strict confidentiality regarding supported mothers.

**Table 2:** Topics in the training manual for peer volunteers

I. INTRODUCTION TO PEER SUPPORT	
a) What is a peer volunteer?	
b) Peer volunteer qualities, skills, and expectations	
c) Peer volunteer role, discussion themes, and frequently	
II: BENEFITS OF PEER SUPPORT	
a) emsp;Mothers Helping Mothers Model	
b) Emotional, informational, and validation/appraisal support	
c) Benefits of peer support	
III: RELATIONSHIP DEVELOPMENT	
a) Getting connected	
b) Staying connected	
c) Developing a relationship with the mother	
IV: TECHNIQUES FOR EFFECTIVE TELEPHONE SUPPORT	
a) Empathetic listening and reflection	
b) Using open-ended questions	
c) Listening to more than the words	
d) Problem-solving and exploring options	
V: GENERAL POSTPARTUM DEPRESSION INFORMATION	
a) What is postpartum depression?	
b) Incidence, symptoms, and different presentation of postpartum depression	
c) Causes of postpartum depression	
d) Detection and treatment of postpartum depression	
VI: THE HELPING PROCESS	
a) The relationship between feelings, behaviors, and thoughts	
b) The Three-Step Helping Process	
c) Helping the mother to develop a support system	
d) Common maternal difficulties	
VII: REFERRAL TO PROFESSIONAL SERVICES	
a) What constitutes and emergency	
b) Child abuse, child neglect, domestic violence	
c) Possible mental health professional referral sources	
d) What the partner can do	

#### Step 4: screen peer volunteers

The training workshops provided an opportunity to select applicants who were best suited to provide peer support with postpartum mothers. Applicants whose communication skills were deemed inadequate, who demonstrated difficulties participating in discussions about PPD, or who showed evidence of unresolved PPD were excluded from the peer support program. The screening of peer volunteers was done only at the training session. No objective measure of PPD was included, however, this could easily be incorporated. Parity was not considered part of the matching criteria and we matched volunteers based on ethnicity only if this was the mother’s preference. More than half of the volunteers identified themselves as an immigrant but were able to speak to English. Of those who attended the training, 175 (85.8%) were accepted as peer volunteers and matched with at least one new mother in the trial.

### Phase II: implement the intervention

#### Step 5: recruit postpartum women to participate in the program

In Ontario, most women who have given birth receive a telephone call from a public health nurse, usually within the first 24 to 48 hours after hospital discharge. Between November 2004 and September 2006, this routine telephone call was also used to introduce mothers to the peer support program in the participating health regions. During the routine telephone call, public health nurses screened all consenting mothers with the EPDS. Mothers who scored >9 on the EPDS and provided verbal consent were then contacted by a trial coordinator who assessed each woman for eligibility. Inclusion criteria were: (a) live birth; (b) discharged from hospital; (c) <2 weeks postpartum; (d) scored >9 on the EPDS; and (e) availability of a peer volunteer in the mother’s geographical area. There were no eligible mothers enrolled in an area unless there was a peer volunteer available. Exclusion criteria were: (a) infant not discharged home with mother and (b) current use of anti-depressant or anti-psychotic medication. Women with prior PPD were not excluded.

After ensuring eligibility, the trial coordinator provided interested mothers with a detailed program explanation. This program was provided within the context of a randomized controlled trial designed to evaluate the effectiveness of preventing PPD. Consequently, while all mothers in the study had access to the standard community postpartum services, mothers who were randomly allocated to the peer support group also received telephone-based support from a peer volunteer [25]. Of the 349 mothers randomized to the peer support group in the trial, the majority were married (*n* = 323, 93%) and had some postsecondary education (*n* = 279, 79.9%); 40.7% (*n* = 142) were multiparous and 20.6% (*n* = 72) self-reported their nationality as non-Canadian.

#### Step 6: initiate contact between peer volunteers and postpartum women

The trial coordinator provided each mother’s name, telephone number, and address to the peer volunteer coordinator who matched the mother and peer volunteers based on maternal residency – a known effective strategy [26,27]. Peer volunteers were asked to initiate telephone contact with the mothers within 48 to 72 hours of program entry and then as frequently as the two deemed necessary.

#### Step 7: provide peer support

The peer volunteers were asked to make a minimum of four telephone contacts with a mother. The duration, structure, and content of the contacts were individualized based upon maternal desire and need. This type of individualized care has been proven to be effective in the breastfeeding peer support trial, in which mothers who received peer support breastfed significantly longer and more exclusively than mothers without peer support, regardless of the duration and intensity of the support provided [27]. The pilot trial evaluating the effect of peer support on PPD symptomatology also showed individualized care to be feasible and acceptable [26].

Out of 349 mothers in the peer support group, clear documentation of the intervention was found with 328 (94%) mothers. To ensure intervention initiation, the peer volunteer coordinator would contact the peer volunteer within one week of the match. There was no suggested number of mothers a peer volunteer should support at one time; this was based on the peer volunteer. On average, peer volunteers supported two new mothers during the trial (mean = 1.87, SD = 1.50), with a range from one to seven. Peer volunteers were also not required to commit to support a certain number of mothers. We did not want to burden the peer volunteers – some would like the experience and some would not and they were able to stay in the program as long as they followed our protocols. Protocols were developed to provide some standardization to the intervention – all peers were expected to call the mother within 48 to 72 hours of being matched and then to call to the mother four times in total to try and make a connection. If the mother did not respond to any of the attempted contacts then the peer volunteer was to stop calling the mother.

**Figure 1:**
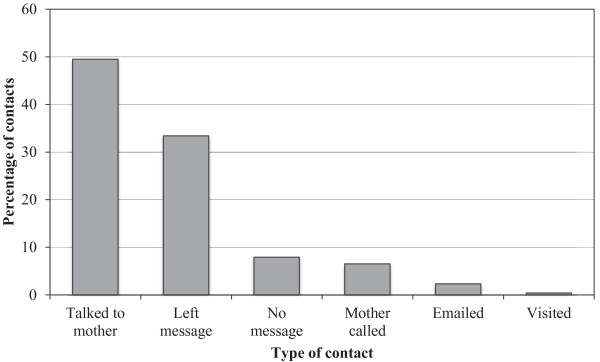
Peer volunteer contacts.

Based on activity logs completed by peer volunteers, mothers received a mean of 8.8 (SD = 6.0) contacts with their peer volunteers. Half of the contacts (*n* = 951, 49.5%) were telephone conversations initiated by the peer volunteer; the mean duration of these discussions was 14.1 minutes (*SD* = 18.5), and ranged from 1 to 180 minutes. Six hundred and forty-one (33.4%) messages were left on mothers’ answering machines; peer volunteers also telephoned mothers but were unable to leave a message (*n* = 151; 7.9%). Of the 1921 peer volunteer-mother contacts, 124 (6.5%) were initiated by the mothers, 8 (0.4%) were face-to-face visits, and 45 (2.3%) were email interactions. These results are depicted in Figure 1 Some mothers were never reached and only received the four telephone messages as required by the program. They were given the peer volunteer’s telephone number and could call the peer volunteer after the peer stopped calling. Peer volunteers were trained how to end a relationship, but the termination time was determined by the mother. The mother could end the peer support at any time. Almost one-third (*n* = 95, 29%) of all peer volunteer-mother matches actively continued past the 12 weeks for which the intervention was monitored [25].

Messages were left on answering machines and this was perceived to be an important part of the intervention as it let the mother know someone cared about her and was available for support. Some peer volunteers had difficulties contacting the mother and this was expected given it was early in the postpartum period. No mothers complained about difficulties contacting a peer volunteer. Some peer volunteers felt hurt when the mother never returned their calls, so in the training session we informed peer volunteers that this may happen and to not take it personally – we asked them to remember what it was like for them early in the postpartum period and how busy it can be.

### Phase III: maintain the intervention

#### Step 8: monitor the intervention initiation and implementation

All intervention activities (such as telephone discussions and left messages) were documented by the peer volunteers using a Peer Volunteer Activity Log [26,27]. Peer volunteers were requested to return the activity log for a supported mother at 12 weeks postpartum. Honoraria were offered to peer volunteers who returned the activity logs. The peer volunteers were not compensated in any other way. A focus group with the pilot peer volunteers suggested they found completing the logs easy and that it promoted consistency and organization in the provision of support. While monitoring of the intervention ceased at 12 weeks, relationships that continued past this time period were indicated on the activity log before it was returned via mail.

#### Step 9: support the peer volunteers

During the training session, peer volunteers were encouraged to incorporate regular consultation with each other. To facilitate this, a listserv was developed and they were given the telephone numbers of the other peer volunteers attending their training session. A newsletter was also distributed to provide trial progress and encouragement. The volunteer coordinator further assisted the peer volunteers in assessing any potential maternal difficulties and developing problem-solving strategies. This assistance was provided via telephone or email on an as-needed basis; however, this did not occur frequently. Of the 121 (69.1%) peer volunteers who completed their evaluation forms, 113 (93.4%) felt that they themselves had enough support. However, 38 peer volunteers would have liked some elements of the peer volunteer program to have been done differently. Examples of these differences that were related to ongoing support included establishing regular meetings for the volunteer and offering opportunities for refresher training or more in-depth training [29].

#### Step 10: review maternal safety and negative effects of the intervention

Because of the potential severity of PPD, several protocols to ensure maternal and child safety were developed and there was regular monitoring for possible negative effects of the intervention. At the baseline screening, any mother who had a positive response on the self-harm ideation item of the EPDS was further assessed by a trained public health nurse within 24 hours. Moreover, any mother who scored ≥20 on any trial EPDS assessment (baseline, 12 weeks, or 24 weeks) or who were diagnosed with major depression at the 12 week or 24 week follow-up assessment were visited by a trained public health nurse to outline treatment options. Less than 5% of mothers scored ≥20 on the EPDS at baseline and they were not recruited. Once a mother was enrolled in the trial, the peer support intervention was not stopped until the mother indicated she wanted it to discontinue. About 5% of mothers were referred to the local public health department due to safety concerns. The public health nurses then referred mothers to a psychiatrist or clinical psychologist as appropriate, based on clinical judgment.

Negative effects of the intervention were assessed through maternal and peer volunteer evaluations as well as the Peer Volunteer Activity Log. The trial coordinator regularly reviewed these data; consistently non-compliant peer volunteers (*n* = 4) were released from the program. The Peer Support Evaluation Inventory [26] included several items related to negative effects of the intervention such as: (a) ‘“my peer minimized my problems’; (b) ‘my peer would get over-involved in my problems’; (c) ‘my peer pressured me to change’; (d) ‘my peer was critical of me’; and (e) ‘my peer made me feel angry’. It was very rare for a mother to report a positive response to these items [30]. Peer support programs should include mechanisms by which negative effects can be identified and prevented in future peer volunteers through revised training sessions.

### Phase IV: evaluate the peer support program

#### Step 11: evaluation of the telephone support program by peer volunteers

When a peer volunteer discontinued providing support or at the completion of the program, a Peer Volunteer Experience Questionnaire [26,27] was administered. This questionnaire had been used successfully in previous peer support trials and includes questions related to: (a) program training; (b) mother-volunteer interactions; (c) volunteer roles; (d) personal effects; and (e) recruitment and retention. In general, peer volunteers felt positively about their participation in the program. Overall, 91.5% of peer volunteers answered yes to the question: ‘If you could do it over again, would you become a peer volunteer?’. Peer volunteer perceptions of their experience have been published elsewhere [29].

#### Step 12: evaluation of the telephone support program by participants

Social support experts have recommended a comprehensive analysis of supportive interactions in order to promote theoretical understanding and the development of more effective interventions [31]. We used the validated Peer Support Evaluation Inventory [26] to provide an in-depth assessment of maternal perspectives of the program at 12 weeks postpartum. The inventory is a four subscale self-report instrument developed to measure an individual’s perception of support received from a peer. The four subscales assessed supportive interactions, relationship qualities, perceived benefits, and satisfaction with support. Cronbach’s alphas for all four subscales were above 0.91 [26]. Two hundred and twenty-one (63.3%) women in the intervention group returned their mailed evaluations of their peer volunteer experiences. Overall, 80.5% of these women were satisfied with their experience, with 83% agreeing that they would recommend this type of support to a friend and 72.2% stating that they felt that their peer provided the assistance that they needed. Maternal perceptions of their peer support experience have been published elsewhere [30].

## Discussion

This paper is the first to document the process of developing, initiating, maintaining, and evaluating a peer support program that is effective for the prevention of PPD. Overall, the program was feasible and had high levels of satisfaction from both peer volunteers and mothers. The program was conducted as the intervention arm in a randomized controlled trial. As such, we were able to hire a trial coordinator who was responsible for all participant recruitment activities, a volunteer coordinator who was responsible for all peer volunteer activities, including training and recruitment of the volunteers and matching of volunteers to participants, and two research nurses who were responsible for all data collection. A cost-effectiveness analysis of this program has been completed [32].

This intervention is very cost-effective when one considers the cost of treating the consequences of PPD, especially among children. However, programs with fewer resources may have different challenges and different results than those seen in the context of a clinical trial.

In our setting, we were able to piggyback our program onto an existing public health system that telephones most new mothers within the first week postpartum after hospital discharge and provides screening for depressive symptoms. Communities which lack similar programs may need to develop different techniques for identification of these high-risk mothers, such as screening at hospital discharge or at the first follow-up visit with a health professional. Alternatively, using the framework that we have described, new programs can be developed which integrate peer support as an important component of postpartum health promotion efforts.

## Conclusions

Peer support, within the health care context, is the provision of emotional, appraisal, and informational assistance by created social network members who possess experiential knowledge of a specific stressor and similar characteristics as the target population, to address a health-related issue of a potentially or actually stressed focal person [28]. This paper describes how a telephone-based peer support program was developed and implemented to prevent PPD among high-risk women. It may be an effective intervention that reduces the risk of PPD symptoms at 12 weeks postpartum. Based on our experience and lessons learned from this peer support trial, a pilot trial, and a previous breastfeeding peer support trial, the 4-phase, 12-step approach that has been delineated provides a formula for the development and implementation of a feasible PPD peer support program that can be adapted for other communities. Moreover, this approach can be used in the development of peer support interventions for other health problems.

## Abbreviations

PPD: postpartum depression; EPDS: Edinburgh Postnatal Depression Scale.

## Competing interests

The author declares no competing interests.

## Authors’ information

C-LD (PhD) is a Professor at the University of Toronto and Senior Scientist at the Women’s College Research Institute in Toronto, Canada. She holds a Canada Research Chair in Perinatal Community Health at the University of Toronto and the Shirley Brown Chair in Women’s Mental Health Research at Women’s College Research Institute.
